# Climate Change Effects on Heat- and Cold-Related Mortality in the Netherlands: A Scenario-Based Integrated Environmental Health Impact Assessment

**DOI:** 10.3390/ijerph121013295

**Published:** 2015-10-23

**Authors:** Maud M. T. E. Huynen, Pim Martens

**Affiliations:** International Centre for Integrated Assessment and Sustainable Development (ICIS), Maastricht University, P.O. Box 616, 6200-MD Maastricht, The Netherlands; E-Mail: p.martens@maastrichtuniversity.nl

**Keywords:** adaptation, climate change, cold, health, heat, mortality, temperature, scenarios

## Abstract

Although people will most likely adjust to warmer temperatures, it is still difficult to assess what this adaptation will look like. This scenario-based integrated health impacts assessment explores baseline (1981–2010) and future (2050) population attributable fractions (PAF) of mortality due to heat (PAF_heat_) and cold (PAF_cold_), by combining observed temperature–mortality relationships with the Dutch KNMI’14 climate scenarios and three adaptation scenarios. The 2050 model results without adaptation reveal a decrease in PAF_cold_ (8.90% at baseline; 6.56%–7.85% in 2050) that outweighs the increase in PAF_heat_ (1.15% at baseline; 1.66%–2.52% in 2050). When the 2050 model runs applying the different adaptation scenarios are considered as well, however, the PAF_heat_ ranges between 0.94% and 2.52% and the PAF_cold_ between 6.56% and 9.85%. Hence, PAF_heat_ and PAF_cold_ can decrease as well as increase in view of climate change (depending on the adaptation scenario). The associated annual mortality burdens in 2050—accounting for both the increasing temperatures and mortality trend—show that heat-related deaths will range between 1879 and 5061 (1511 at baseline) and cold-related deaths between 13,149 and 19,753 (11,727 at baseline). Our results clearly illustrate that model outcomes are not only highly dependent on climate scenarios, but also on adaptation assumptions. Hence, a better understanding of (the impact of various) plausible adaptation scenarios is required to advance future integrated health impact assessments.

## 1. Introduction

Managing the health effects of temperature in response to climate change is a global public health challenge. The Intergovernmental Panel on Climate Change (IPCC) [1] clearly states that it is extremely likely that human influence has been the dominant cause of the observed temperature increase since the mid-20th century and that the continued emissions of greenhouse gasses will cause further warming over the coming decades. The expected increase in global mean surface temperature ranges between 0.3 and 4.8 °C by 2100 (relative to 1986–2005) [1]. This prospect of further global warming is accompanied by increasing concerns about its health implications, including direct impacts such as heat stress and flooding, indirect impacts mediated through natural systems such as infectious diseases and air quality (including aeroallergens such as pollen), and impacts heavily mediated by human systems such as food security [2,3,4,5,6]. This growing awareness of climate change health impacts was recently highlighted by the publication of the reports of the Lancet Commission on Health and Climate Change [7] and the Rockefeller Foundation–Lancet Commission on Planetary Health [8].

Within the Netherlands, the direct health effect of temperature under a changing climate has been identified—by the Health Council of The Netherlands, the Dutch Court of Audits, policymakers (incl. national government), scientists, health professionals, advocacy groups and other stakeholders—as an important issue demanding further research and policy action [9,10,11,12,13,14,15,16,17]. In response, this paper focuses on the direct effects of future ambient temperature change on mortality in the Netherlands. The projected change in climate is believed to influence both annual (fractions of) cold-related and heat-related mortality [6]. The physiological mechanisms that underlie cold-related mortality are believed to have mainly cardiovascular and respiratory effects; the physiological pathways explaining heat-related deaths seem to include, for example, changes in heart rate, fluid and electrolytic balance, blood viscosity and vasoconstriction [18]. Several factors may modify the association between ambient temperature and mortality over time, including intrinsic biological factors (such as life stage, health status and physiological adaptations) as well as extrinsic factors (such as extensive heat-health warning systems and other public health responses, changes in activity patterns and time spent outside, changes in the built environment, air quality, air conditioning, and greater awareness of the dangers of extreme heat or cold). Hence, it is likely that the temperature–mortality risk function may change in the future as the population will (partially) adapt to higher temperatures; yet it is still difficult to assess what this adaptation will look like. There is, therefore, a need to better understand the changes in (Dutch) cold- and heat-related mortality under different future climate and adaptation pathways [6,10,12,19,20,21].

Traditional methods of health risk assessment have provided good service in support of policy, mainly in relation to standard setting and regulation of hazardous chemicals or practices. In recent years, however, it has become apparent that many of the global environmental risks facing society are set within wider social, economic and environmental contexts and encompass longer time frames [22,23]. As a result, traditional forms of risk assessment do not form an adequate basis for assessment of the systemic and/or longer-term environmental health risks that we currently face, such as global warming. As the assessment of future climate change health impacts is inherently prognostic in nature, a forward looking approach exploring different future scenarios is required. Scenarios can be defined as descriptions of journeys to possible futures that reflect different assumptions about how current trends will unfold, how critical uncertainties will play out and what new factors will come into play [24]. Hence, a scenario-based approach explores the health impacts of (interlinked) developments in a range of possible future pathways in the face of uncertainty. However, in much traditional risk assessment, the role of scenarios has often been implicit rather than explicit [23].

An innovative assessment framework termed integrated environmental health impact assessment (IEHIA) provides a promising way forward; IEHIA is defined as a means of assessing health-related problems deriving from the environment, and health-related impacts of policies and other interventions that affect the environment, in ways that take account of the complexities, interdependencies and uncertainties of the real world [23]. This approach has been elaborated in the EU funded INTARESE project [22,23]. However, even within the INTARESE project, the importance of scenarios for integrated health impact assessment was only recognized during the project [22].

In recent years, the need for an integrated and scenario-based approach in studying the Dutch health impacts of global warming has been widely acknowledged [10,12]. In this study, we will explore a range of possible futures, aiming at answering “what if” questions about future heat- and cold-related mortality in view of climate change, adaptation and increasing overall mortality. We will model baseline (1981–2010) and future (2050) population attributable fractions (PAF) of mortality (total, cardiovascular, respiratory) due to heat (PAF_heat_) and cold (PAF_cold_), by combining observed temperature–mortality relationships with the new Dutch climate scenarios developed by the Royal Netherlands Meteorological Institute (KNMI’14 scenarios) and different adaptation scenarios. Accordingly, the associated attributable numbers of deaths are modelled accounting for the future increase in overall Dutch mortality.

## 2. Methods

**Figure 1:**
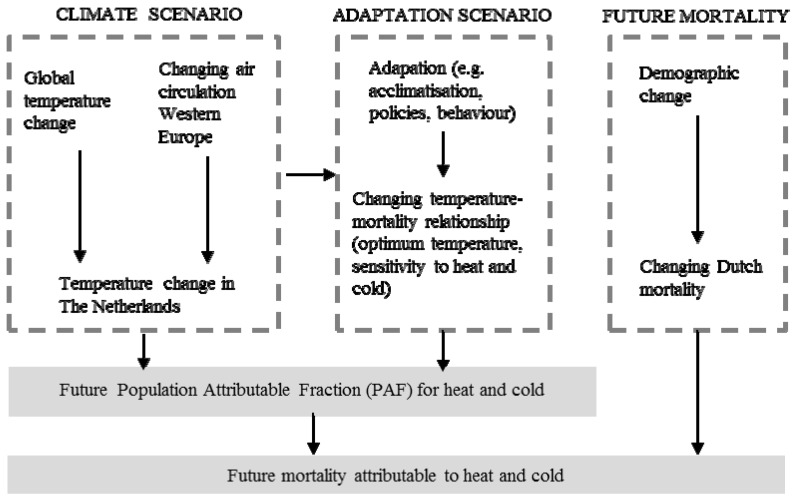
The scenario-based integrated environmental health impact approach underlying this study.

We take a scenario-based integrated environmental health impact assessment approach. The contribution of heat exposure and cold exposure to mortality is quantified using the population attributable fraction (PAF; expressed as percentage of all deaths for the selected cause of death). The PAF-based model underlying this study is based on observed exposure–response functions of temperature and mortality that are applied to a baseline period (1981–2010) and the KNMI’14 scenarios climate scenarios (2050) in order to estimate current and future population attributable fractions of mortality due to exposure to heat and cold. Calculations are performed for total mortality, cardiovascular mortality and respiratory mortality. Several model runs are conducted in order to generate outcomes with and without different adaptation scenarios. In calculating the future number of deaths attributable to heat and cold, we also account for future demographic change (mortality trend). Figure 1 provides an outline of our scenario-based approach.

### 2.1. Observed Temperature–Mortality Relationship

Many studies (e.g., [18,25,26,27,28,29,30,31,32,33]) have shown a “V shape”, or “U-shape”, relationship between temperature and mortality, with mortality rates lower on days with a temperature closer to the temperature level corresponding to the lowest point of the curve (*i.e.*, minimum mortality temperature or optimum temperature). Hence, cold and heat effects are commonly quantified separately, assuming a mostly linear response below and above a threshold temperature [19,20,32,34,35]. The prevailing climate of a geographical area appears to determine the level of this optimum temperature level, as this threshold level is higher in warmer climates, suggesting adaptation [19,32,36]. The slopes of the curve indicate the size of the effect [19]. Generally, heat (or cold) effects are presented as the percentage change in mortality for every 1 °C increase (decrease) in temperature above (below) the heat (cold) threshold.

For this study, the observed exposure–response functions (ERFs) of temperature and mortality are derived from an earlier time series analyses conducted by Huynen *et al.* [37], which focussed on the daily numbers of deaths due to all causes and to selected causes in relation to daily average temperature in the Netherlands, 1979–1997. This period largely overlaps with the baseline period (1981–2010) of the current study. The ERFs are based on a linear threshold model, controlled for season and time trend. Huynen *et al.* [37] found an expected V-like relationship between average temperature and mortality with an optimum average temperature level of 16.5 °C for total mortality, cardiovascular mortality and respiratory mortality. If the average temperature is above this optimum temperature level, people are exposed to heat. If the average temperature is below the optimum temperature level, people are exposed to cold. The level of exposure is measured as the difference between the observed average temperature and the optimum temperature.

The ERF for heat is based on the percentage change in mortality for each degree Celsius increase in the average value of heat exposure (above the optimum temperature level). The ERF for cold is based on the percentage change in mortality for each degree Celsius increase in the average value of cold exposure (below the optimum temperature level). For both heat and cold exposure, lagged effects up to one month are taken into account in order to include the long delay of the effects of cold and to exclude deaths that were advanced by only a few days (harvesting effect) (see also [18]). Huynen *et al.* [37] found that for each degree Celsius increase above the optimum temperature, mortality increased by 2.72% for total mortality, 1.86% for cardiovascular disease, and 12.82% for respiratory diseases. For temperatures below the optimum, mortality in the same categories increased by 1.37%, 1.69% and 5.15% for each degree Celsius decrease. Hence, the percentage mortality change for each degree rise in heat exposure is larger than for each degree rise in cold exposure. The mortality due to malignant neoplasms was found to be less sensitive to temperature [37]; hence, this cause of death has been excluded from the current analysis.

### 2.2. KNMI’14 Climate Change Scenarios

In 2014, a new set of Dutch climate change scenarios was published by the Royal Netherlands Meteorological Institute (KNMI): the so-called KNMI’14 scenarios [38,39]. These scenarios are based on the fact that future climate change in the Netherlands will mainly depend on the global temperature rise as well as on changes in the air circulation patterns in Western Europe. The anticipated changes in the climatological target period are described relative to a climatological baseline period 1981–2010.

To be able to deal with the uncertainties in future climate change, four climate scenarios from the broad range of possible futures were selected [38,39]. The KMMI considers it most likely that the Dutch climate will develop between these four “corner points”, representing a global average temperature increase of 1 °C (G-scenarios) and 2 °C (W-scenarios) around 2050, both with low (L-scenarios) and high values (H-scenario) for the anticipated change in air circulation patterns in Western Europe (Table 1). The anticipated change in air circulation pattern has a substantial influence on future climate change in the Netherlands. In the H-scenarios, more frequent westerly winds occur in winter, leading to mild and more humid weather compared to the L-scenarios. In summer, high-pressure systems have a greater influence on the weather in the H-scenarios, causing more Easterly winds, which implies warmer and drier weather for the Netherlands.

A transformation of historic observations translates the climate change signal derived from the models into future time series at the different Dutch stations [40]. The current study uses the observed and transformed time series of daily temperature at the De Bilt station (located in the centre of the Netherlands) at baseline (1981–2010) and 2050 (2035–2065). Table 1 shows that in all KNMI’14 scenarios the Dutch annual temperature rise (compared to baseline) is higher than the global temperature rise, especially in the scenarios with larger changes in air circulation (KNMI’14-G_H_ and KNMI’14-W_H_). Noteworthy is the fact that in all KNMI’14 scenarios the temperature increase is not distributed evenly throughout the year; e.g., the temperature increase is higher in the winter compared to summer. An elaborate description with all relevant sources and references to the scientific literature can be found in van den Hurk *et al.* [39].

**Table 1:** The KNMI’14 climate scenarios [38,39,40,41].

KNMI’14 Scenario			Mean Temperature Increase in De Bilt (the Netherlands) in 2050, Compared to the Baseline Period 1981–2010 * (per Season **)				
Name ***	Global Temperature Rise on Earth	Change in Air Circulation Patterns in Western Europe	Spring	Summer	Autumn	Winter	Annual
KNMI’14-G_L_	1 °C around 2050 1.5 °C around 2085	Low value	+0.9 °C	+1.1 °C	+1.1 °C	+1.2 °C	+1.1 °C
KNMI’14-G_H_	1 °C around 2050 1.5 °C around 2085	High value ****	+1.2 °C	+1.4 °C	+1.4 °C	+1.8 °C	+1.5 °C
KNMI’14-W	2 °C around 2050 3.5 °C around 2085	Low value	+1.9 °C	+1.8 °C	+2.3 °C	+2.3 °C	+2.1 °C
KNMI’14-W	2 °C around 2050 3.5 °C around 2085	High value ****	+2.1 °C	+2.4 °C	+2.4 °C	+2.8 °C	+2.4 °C

* Mean temperature at baseline (1981–2010): Spring = 9.5 °C; Summer = 17 °C; Autumn= 10.6 °C; Winter = 3.4 °C; Annual: 10.1 °C; ** Spring = March-April-May; Summer = June-July-August; Autumn = September-October-November; Winter = December-January-February; *** Within the scenario classification, G stands for Gematigd (Dutch for moderate) and W stands for Warm (Dutch for warm); **** Milder and wetter winters due to more westerly winds; warmer and dryer summers due to more easterly winds.

### 2.3. Adaptation Scenarios

People will probably adapt to the changing climate over time, at least to some extent [6,19]. Geographical variations in optimum temperature values have been documented, suggesting that populations living in warmer areas have adapted to the higher temperatures [18,20,42,43,44]. Previous studies [45,46,47] assumed that global warming could result in an increase in the optimum temperature value as populations adjust to the warmer climate; the change in this optimum level for each scenario should reflect the rate of warming experienced, and is, therefore, anticipated to be proportional to the projected change in temperature.

Geographical variation is also observed in the sensitivity to heat or cold exposures (*i.e.*, sensitivity to departures from the optimum temperature value), although the factors (e.g., climate, socio-economic and demographic factors) contributing to these geographical differences are not always clear [20]. Adaptive responses (e.g., physiological, air-conditioning, improved housing, and improved air quality) and public health interventions (e.g., heat-health warning systems) might decrease people’s vulnerability to high ambient heat [19]. On the other hand, it has been argued that population sensitivity to cold weather is greater in temperate regions with mild winters, as their populations are less well adapted to cold exposures [48,49,50];hence, the current study includes adaptation scenarios anticipating that the slope of the ERF for cold exposure becomes steeper.

In this study, we will account for different adaptation scenarios, assuming a shift in the optimum temperature level proportional to the anticipated increase in temperature as well as assuming changes in the sensitivity to temperatures above and below the optimum temperature. Hence, we will run our model with and without accounting for three adaptation scenarios (Table 2). Adaptation scenario I represents a shift in the current temperature–mortality relationships to the future, proportional to the anticipated temperature change; for each climate change scenario, the new optimum temperature level is calculated by adding the change in annual average temperature to the baseline optimum temperature level. Adaption scenario II represents a 10% decrease in the sensitivity to heat exposure as summers become warmer and a 10% increase in the sensitivity to cold exposure as winters become milder. Finally, adaption scenario III combines the assumptions made in scenario I and scenario II.

**Table 2:** The adaptation scenarios.

Future Adaptation Scenario	Scenario Assumption	Optimum Temperature (for All Selected Causes)	Sensitivity to Heat (Slope ERF) *	Sensitivity to Cold (Slope ERF) **
No adaptation	Baseline values (based on [37]).	16.5 °C for all KNMI’14 scenarios	2.72% for total mortality, 1.86% for cardiovascular mortality, 12.82% for respiratory mortality	1.37% for total mortality, 1.69% for cardiovascular mortality, 5.15% for respiratory mortality
Adaptation scenario I	Optimum temperature level increases proportional to annual temperature increase	KNMI’14-G_L_: 17.6 °C KNMI’14-G_H_: 18.0 °C KNMI’14-W_L_: 18.6 °C KNMI’14-W_H_: 18.9 °C	2.72% for total mortality, 1.86% for cardiovascular mortality, 12.82% for respiratory mortality	1.37% for total mortality, 1.69% for cardiovascular mortality, 5.15% for respiratory mortality
Adaptation scenario II	10% decrease in sensitivity to heat; 10% increase to sensitivity in cold	16.5 °C for all KNMI’14 scenarios	2.45% for total mortality, 1.67% for cardiovascular mortality, 11.54% for respiratory mortality	1.51% for total mortality, 1.86% for cardiovascular mortality, 5.67% for respiratory mortality
Adaptation scenario III	I and II combined	KNMI’14-G_L_: 17.6 °C KNMI’14-G_H:_ 18.0 °C KNMI’14-W_L_: 18.6 °C KNMI’14-W_H:_ 18.9 °C	2.45% for total mortality, 1.67% for cardiovascular mortality, 11.54% for respiratory mortality	1.51% for total mortality, 1.86% for cardiovascular mortality, 5.67% for respiratory mortality

* Mortality increase for each degree Celsius increase above the optimum temperature; ** Mortality increase for each degree Celsius decrease below the optimum temperature.

### 2.4. Baseline and Future Mortality Data

Baseline and future mortality data were extracted from the online Statline database of Statistics Netherlands [51]. The average annual number of deaths at baseline (1981–2010) was 131,751 for total mortality, 48,881 for cardiovascular mortality and 11,736 for respiratory mortality [52]. According to Statistics Netherlands, the annual number of deaths is anticipated to increase to 200,497 in 2050 [53]. This prognoses is based on the “most likely” demographic trends, including population growth, ageing and a continued declining mortality risk per age group (due to for example medical innovations) [54] No other scenarios of future overall mortality are provided by Statistics Netherlands; no prognoses are provided for mortality due to specific causes [53].

### 2.5. Modelling Population Attributable Fractions of Mortality Due to Heat and Cold Exposure

In order to estimate baseline and future exposure to heat and cold, the daily temperature difference above (heat) respectively below (cold) the optimum temperature level are calculated for the baseline time series, as well as the transformed time series for each KNMI’14 scenario. The resulting current and future exposures to heat/cold are, accordingly, applied to the ERF for heat/cold in order to estimate the current and future annual PAFs due to exposure to heat/cold.

As it is assumed that the whole population is exposed, the PAF is similar to the attributable risk percentage (AR% = percent of deaths in the exposed population that is due to the exposure) [55]. Hence, the PAF_heat_, PAF_cold_ and PAF_temperature_ are modelled as follows:

*Population attributable fraction (%) of mortality due to heat exposure (Equation (1)):*

(1) PAF_heat_ = (RR_heat_ − 1)/RR_heat_ × 100%

where

RR_heat_ = exp(β_heat_ × E_heat_)
β_heat_ = ln[(ERF_heat_/100) + 1]
ERF_heat_ = % change in mortality per 1 °C increase in E_heat_
E_heat_ = temperature exposure above the threshold/optimum temperature (in °C)

*Population attributable fraction (%) of mortality due to cold exposure (Equation (2)):*

(2) PAF_cold_ = (RR_cold_ − 1)/RR_cold_ × 100%

where

RR_cold_ = exp(β_cold_ × E_cold_)
β_cold_ = ln[(ERF_cold_/100) + 1]
ERF_cold_ = % change in mortality per 1 °C increase in E_cold_
E_cold_ = temperature exposure below the threshold/optimum temperature (1 °C)

*Net population attributable fraction (%) of mortality due to heat and cold exposure (Equation (3)):*

(3) PAF_temperature_ = PAF_heat_ + PAF_cold_

The PAF-calculations are performed for the baseline period as well as the KNMI’14 scenarios (2050), with and without adjustment for adaptation, and for all selected causes (total mortality, cardiovascular mortality, respiratory mortality).

### 2.6. Modelling the Number of Deaths Attributable to Heat and Cold Exposure

In order to translate the PAFs into number of deaths attributable to baseline and future exposure to heat and cold, the modelled PAFs are, subsequently, combined with baseline and future mortality data. The estimated numbers of deaths attributable to exposure to heat and cold are modelled as follows:

*Number of deaths attributable to heat exposure (Equation (4)):*

(4) M_heat_ = PAF_heat_ × m

where m is the number of annual deaths

*Number of deaths attributable to cold exposure (Equation (5)):*

(5) M_cold_ = PAF_cold_ × m

where m is the number of annual deaths

*Net number of deaths attributable due to heat and cold exposure (Equation (6)):*

(6) M_temperature_ = M_heat_ + M_cold_

The 2050 mortality prognoses are not available for specific causes. Hence, future attributable deaths due to heat and cold exposure are only calculated for total mortality. In order to differentiate between the future attributable deaths with and without a changing climate, we apply both the future PAF estimates (based on future exposure to heat and cold according to the KNMI’14 scenarios) and the baseline PAF estimates (based on baseline exposure to heat and cold) to the future number of annual deaths.

## 3. Results

This section first describes the estimated baseline and future exposure levels to heat and cold, with and without adaptation. Subsequently, the resulting model estimates for the baseline and future PAFs of mortality due to exposure to heat and cold are discussed. Finally, the associated baseline and future attributable deaths (total mortality) are provided.

### 3.1. Baseline and Future Exposure to Heat and Cold

Table 3 shows the baseline and future exposures to heat and cold for the Dutch KNMI’14 climate scenarios, with and without the shift in optimum temperature level (see also Table 2). During the baseline period, average daily exposure to heat was 0.43 °C, while the average exposure to cold was 6.85 °C (most of the average daily temperatures being lower than the optimum value).

**Table 3:** Baseline (1981–2010) and future (around 2050) average daily exposure to heat and cold, according to the KNMI’14 scenarios, with and without a changing optimum temperature level.

Climate Scenario	Adaptation Assumption Regarding Optimum Temperature Level in 2050			
	No Shift in Optimum Temperature Compared to Baseline *		Optimum Temperature Level Increases Proportional to Annual Temperature Increase **	
	Exposure to Heat	Exposure to Cold	Exposure to Heat	Exposure to Cold
Baseline	0.43	6.85	0.43	6.85
KNMI’14-G_L_	0.62	6.01	0.42	6.90
KNMI’14-G_H_	0.70	5.72	0.42	6.93
KNMI’14-W_L_	0.82	5.20	0.39	6.87
KNMI’14-W_H_	0.95	4.99	0.43	6.86

* This assumption is applied in the 2050 model runs without adaptation and the 2050 model runs applying adaptation scenario II; ** This assumption is applied in the 2050 model runs applying adaptation scenario I and the 2050 model runs applying adaptation scenario III.

The optimum temperature remains unchanged at its baseline level in the 2050 model runs without adaptation and the 2050 model runs applying adaptation scenario II. In this case, heat exposure increases with 0.19 °C in KNMI’14-G_L_, 0.27 °C in KNMI’14-G_H_, 0.39 °C in KNMI’14-W_L_, and 0.52 °C in KNMI’14-W_H_, while cold exposure decreases with 0.84 °C in KNMI’14-G_L_, 1.13 °C in KNMI’14-G_H_, 1.65 °C in KNMI’14-W_L_, and 1.86 °C in KNMI’14-W_H_ (compared to baseline; Table 3). Both the changes in heat and cold exposure are larger in KNMI’14-G_H_ and KNMI’14-W_H_ compared to KNMI’14-G_L,_ respectively KNMI’14-W_L_, because the altering air circulation patterns result in hotter summers as well as milder winters.

Table 3 reveals that, for each KNMI’14 scenario, the shifting optimum temperature in adaption scenarios I and III results in a lower heat exposure and a higher cold exposure compared to the 2050 model runs without this adaption assumption. However, it is important to note that, while this shift in optimum temperature is assumed to be proportional to the annual temperature increase, this annual temperature increase is not evenly distributed throughout the year (see also Table 1). Hence, applying adaption scenarios I and III results in a slight decrease in heat exposure compared to the baseline (as the increase in summer temperatures is lower than the annual average). As the increase in winter temperatures is higher than then the average annual change, one might expect a decrease in cold exposure under adaptation scenarios I and III. However, applying a shift in optimum temperature proportional to the average annual temperature increase results in an increase in cold exposure for each KNMI’14 scenario compared to the baseline (Table 3). This seems surprising, but can be explained by the relative lower increases in spring and autumn temperatures (exposure to cold is not restricted to the winter season, but is also common in spring and autumn).

### 3.2. Population Attributable Fractions of Mortality Due to Heat and Cold

**Figure 2:**
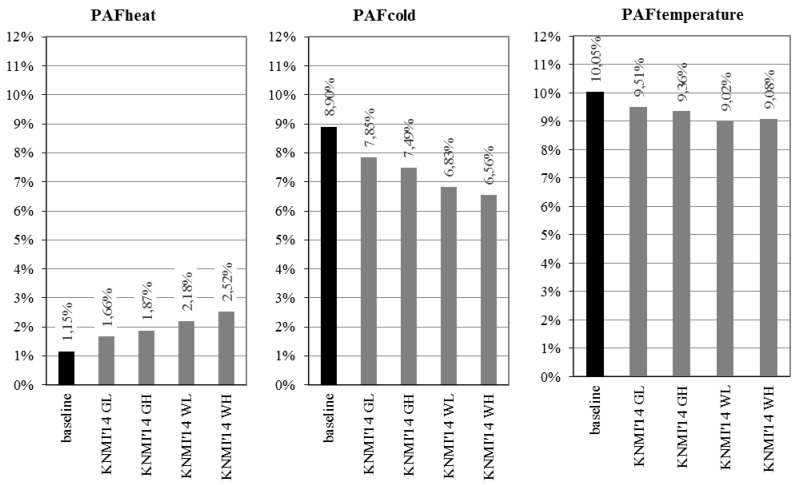
Total mortality—Population attributable fractions (PAF) of mortality due to exposure to heat and cold, at baseline (1981–2000) and in 2050 (KNMI’14 scenarios), the Netherlands: model runs without adaptation.

The baseline model estimates show that for total mortality the PAF_heat_ is 1.15%, the PAF_cold_ is 8.90% and the PAF_temperature_ is 10.05%. In case of no adaptation, the 2050 model results for total mortality indicate that PAF_temp_ decreases in all KNMI’14 scenarios (to 9.02%–9.51% in 2050), as the decrease in PAF_cold_ (to 6.56%–7.85% in 2050) outweighs the increase in PAF_heat_ (to 1.66%–2.52% in 2050). Figure 2 further illustrates these developments (Table A1 provides a detailed overview of all model run outcomes).

**Figure 3:**
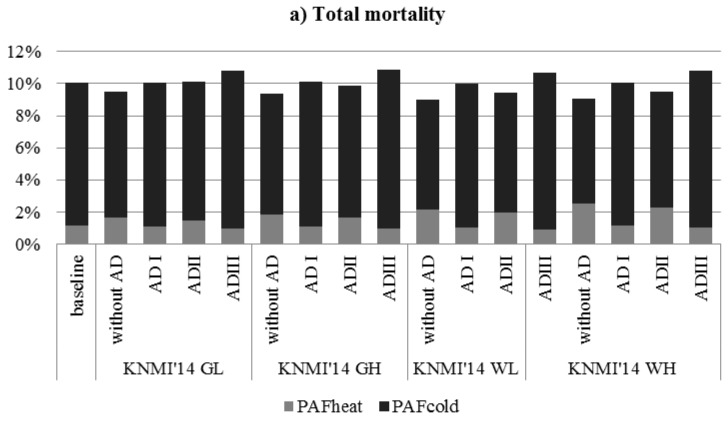

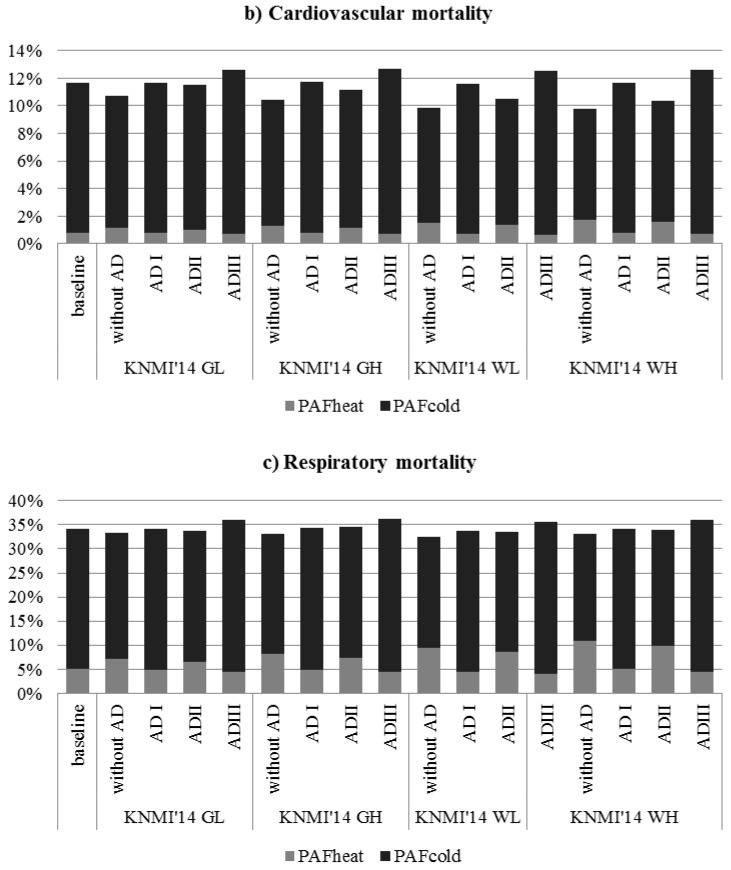
Population attributable fractions (PAF) of mortality (total, cardiovascular, respiratory) due to exposure to heat and cold, at baseline (1981–2000) and in 2050 (KNMI’14 scenarios), the Netherlands: model runs with and without adaptation. Note: without AD = without adaptation; AD I = adaptation scenario I (shift in optimum temperature); AD II = adaptation scenario II (changing sensitivity to heat and cold); AD III = adaptation scenario III (adaptation scenarios I and II combined).

Figure 3 illustrates the PAF_heat_, PAF_cold_ and PAF_temperature_ for total mortality in 2050 according to KNMI’14 climate scenarios, but now accounting for the different adaptation scenarios as well (Table A1 provides a detailed overview of all model run outcomes). Depending on the climate scenario and adaption scenario applied (including the option of no adaptation), the 2050 PAF_heat_ will range between 0.94% and 2.52%, the 2050 PAF_cold_ between 6.56% and 9.85%, and the 2050 PAF_temp_ between 9.02% and 10.85%. A closer look at the different model run outcomes shows that the different adaptation scenarios have different effects on the changes in PAF_heat_ and PAF_cold_. The model results applying adaption scenario I (shift in optimum temperature) show a decrease in PAF_heat_ and a slight increase in PAF_cold_ (compared to the baseline period). As explained above, this results from the uneven distribution of temperature increase throughout the year (while the shift in optimum temperature is assumed to be proportional to the annual temperature increase). The model results applying adaptation scenario II (decreasing sensitivity to heat; increasing sensitivity to cold) show an increase in PAF_heat_ and a decrease in PAF_cold_ compared to the baseline period, but—as expected—these changes are smaller compared to 2050 model runs without adaptation. The 2050 model results applying adaptation scenario III (combining adaptation scenarios I and II) show a decrease in PAF_heat_ and an increase in PAF_cold_ compared to the baseline period.

Similar conclusion can be drawn for cardiovascular mortality and respiratory mortality (Figure 3; Table A2 and Table A3). For cardiovascular mortality, PAF_heat_ is 0.79% and PAF_cold_ is 10.85% at baseline; in 2050 the PAF_heat_ ranges between 0.64% and 1.74% and the PAF_cold_ ranges between 8.01% and 11.99% (depending on climate and adaptation scenario). For respiratory mortality, PAF_heat_ is 5.05% and PAF_cold_ is 29.11% at baseline; in 2050 the PAF_heat_ ranges between 4.16% and 10.86% and the PAF_cold_ ranges between 22.15% and 31.76%.

### 3.3. Baseline and Future Mortality Due to Heat and Cold Exposure

**Figure 4:**
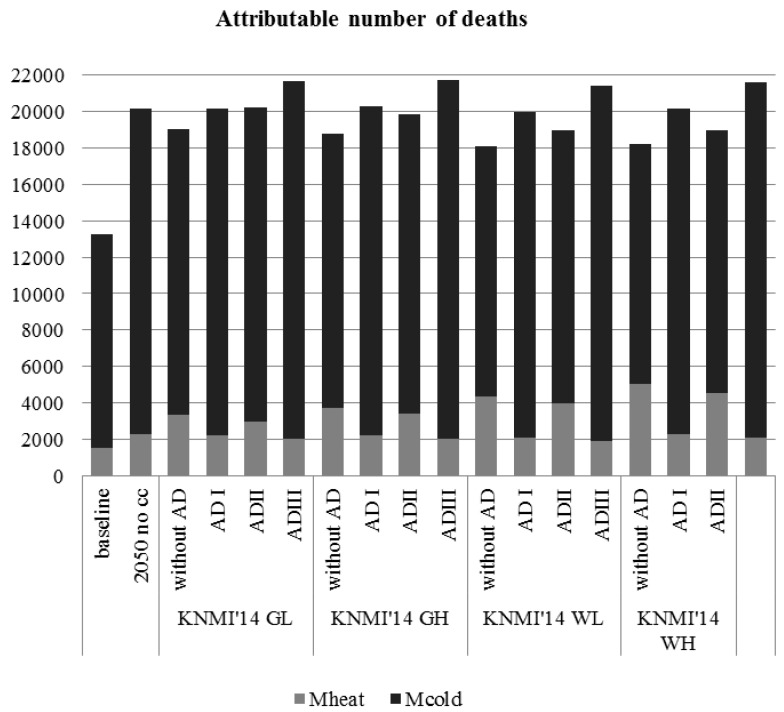
Baseline and future number of deaths attributable to heat (M_heat_) and cold (M_cold_), at baseline (1981–2000) and in 2050 (KNMI’14 scenarios), the Netherlands: model runs with and without adaptation. Note: 2050 no cc (no climate change) = 2050 model runs applying baseline attributable fractions to future number of annual deaths; without AD = without adaptation; AD I = adaptation scenario I (shift in optimum temperature); AD II = adaptation scenario II (changing sensitivity to heat and cold); AD III = adaptation scenario III (shift in optimum temperature as well as changing sensitivity to heat and cold).

The modelled number of temperature-related deaths per year (taking the baseline/future mortality into account) are shown in Figure 4 (Table A4 provides a detailed overview of all model run outcomes). The 2050 model outcomes reveal that climate change in the absence of adaptation results in an increasing heat-related mortality (M_heat_) ranging between 3329 and 5061 deaths in 2050 (depending on the KNMI’14 scenario), from a baseline of 1511 deaths. Cold-related mortality (M_cold_) will increase to 13,149–15,733 deaths in 2050, from a baseline of 11,727 deaths. When the 2050 model runs applying the different adaptation scenarios are considered as well, however, the M_heat_ ranges between 1879 and 5061 deaths, and the M_cold_ ranges between 13,149 and 19,753 deaths. It is important to note that these changes result from the combined effect of an increasing number of annual deaths and changing heat and cold exposures. Additionally, Table A4 provides the percentage change in attributable mortality compared to the baseline.

In case of no climate change (applying the baseline PAF_heat_ and PAF_cold_ to the future mortality), future M_heat_ is 2299 and M_cold_ is 17,846. The difference between the mortality burdens with and without climate change reflects the change in M_heat_ and M_cold_ that are due to the future temperature increase (see Table A4).

## 4. Discussion

This scenario-based integrated health impacts assessment explores Dutch baseline (1981–2010) and future (2050) population attributable fractions (PAF) of mortality (total, cardiovascular, respiratory) due to heat (PAF_heat_) and cold (PAF_cold_), by combining observed temperature–mortality relationships with the national KNMI’14 climate scenarios and three adaptation scenarios. As we focus on exposure to non-optimum temperatures only, we did not consider the mortality effects of extreme cold or hot days. Gasparini *et al.* [18] argue that most deaths are caused by exposure to moderately hot and cold temperatures, and the contribution of extreme days is comparatively low.

Our results reveal that the baseline PAF_heat_ is 1.15% and the baseline PAF_cold_ is 8.90%. Other studies reporting attributable risk measures for whole-year mortality find values relative close to ours, provided the different analytical approaches. Hajat *et al.* [56] found that mortality attributable to heat ranged between 0.37% and 1.45% in three European cities (London, Budapest and Milan). For London, Carson *et al.* [57] found that 5.4% of deaths were attributable to cold but none to heat. In a recent large multicountry study (based on data for 384 locations in Australia, Brazil, Canada, China, Italy, Japan, South Korea, Spain, Sweden, Taiwan, Thailand, UK, and USA), Gasparini *et al.* [18] found that the mortality attributable to cold was 7.29% (7.02%–7.49%) and mortality attributable to heat was 0.42% (0.39–0.44).

Our model outcomes without adaptation reveal that climate change results in a decrease in PAF_cold_ (6.56%–7.85% in 2050) that outweighs the increase in PAF_heat_ (1.66%–2.52% in 2050). When the 2050 model runs applying the different adaptation scenarios are considered as well, however, the PAF_heat_ ranges between 0.94% and 2.52% and the PAF_cold_ between 6.56% and 9.85%. Hence, depending on the adaptation scenario, the PAF_heat_ can decrease or increase compared to the baseline. The same holds for PAF_cold_. Similar conclusions can be drawn from the model outcomes for cardiovascular mortality and respiratory mortality. Our estimates of the associated 2050 mortality burdens—accounting for both the temperature increase and the overall increase in Dutch mortality—show that the number of heat-related deaths ranges between 1879 and 5061 (1511 at baseline) and the number of cold-related deaths ranges between 13,149 and 19,753 (11,727 at baseline). Without climate change, our 2050 model outcomes show 2299 heat-related deaths and 17,846 cold related deaths in 2050 (due to increasing mortality trend only).

Although people will most likely adjust to a warmer climate, we need to recognise the significant uncertainty over the degree of physiological, social, or technological adaptation over long time periods [6]. Due to this uncertainty, however, past studies into future temperature-related mortality impacts assumed in their models that no adaptation would occur. For example, Baccini *et al.* [58] argued that epidemiological evidence of the extent to which short- or long-term adaptation alters mortality risk is limited and sometimes contradictory. For their model projections, these authors, therefore, assumed that no acclimatization occurred, and hence there would be no future change in the temperature–mortality relationship. They acknowledged, however, that this assumption probably conveyed a degree of overestimation of the impact of warmer summers in the future. Other studies [59,60,61] also assumed no future adaptation. A recent UK study by Hajat *et al.* [21] stated that its main focus was on estimating the contribution of climate change on future mortality burdens in the absence of other changes, and so these possible changes in the risk function due to adaptation were not considered. However, the authors did recognize that further work should focus on the modelling of adaptation to rising temperatures.

In two of the three adaption scenarios, the current study accounts for adaptation by shifting baseline temperature–mortality relationships into the future resulting in an increasing optimum temperature level increases with time. This assumption is based on observed geographical variations in optimum temperature levels. For example, Baccini *et al.* [58] argue that the variation from 23–32 °C in city-specific thresholds (March-September period) among 15 European cities reflects population adaptation (e.g., physiologic and behavioral) to the diverse climates across Europe. McMichael *et al.* [20] also concluded that the observed geographical variations in heat thresholds in their study among 12 urban populations in low- and middle-income countries could be explained by the in difference in summer temperature, reflecting the adaptation of the population. Previous studies [45,46,47] have applied a shift in optimum temperature to account for climate adaptation, but without making any further assumptions regarding changes in the sensitivity to temperatures above and below this optimum temperature level (*i.e.*, the slope of the temperature–mortality relationship remains unchanged).

However, in the future people might become less vulnerable to exposure to heat; adaptive responses (e.g., air-conditioning, improved housing, and improved air quality) and public health interventions (e.g., heat-health warning systems-HHWS) can affect the future exposure–response relationship between mortality and warm temperature. Huang *et al.* [19] identify two approaches that have been used to account for adaptation and that do reflect such change in vulnerability to heat exposure as well. One approach is based on applying the temperature–mortality curves from analogue cities/countries that represent the future climate of the target city/country. However, there might be methodological differences in estimating the associations, as well as important differences in confounding factors. Using this approach, Knowlton *et al.* [62] estimated for New York that acclimatization may reduce the impact of added summer heat in the 2050s by roughly 25%. For the Netherlands, a temperature–mortality curve from an analogue region using similar methodologies is not available. The other approach is the use of the temperature–mortality curve based on only a selection of analogue warmer-than-normal years for the target city/country, representing short-term (e.g., physiological, behavioural) adaptation based on a rather limited amount of observational data. A possible third approach for estimating changing vulnerability could possibly be derived from the observed effects of adaptation measures. However, there is no conclusive evidence yet about the effectiveness of, for example, Heat Health Warning Systems (HHWS). An Italian study [63] observed a reduction in high temperature’s effect on mortality in the elderly, which was partly attributed to the implementation of heat prevention activities. This decrease, however, was only observed in correspondence with very high temperatures. A recent review by Toloo *et al.* [64] supported the notion that HHWS are effective in reducing heat-related mortality, but also concluded that the number of studies on this topic is limited and that more robust research into the effectiveness of HHWS is needed. Considering the above, it is still difficult to formulate adaptation scenarios that represent evidence-based changes in sensitivity to heat exposure in the Netherlands. It is, however, generally believed that this sensitivity will likely decrease [19]. Hence, we included the assumption that vulnerability to heat will decrease with a hypothetical 10% in our adaptation scenarios.

Additionally, an interesting question to ask is how the sensitivity to cold will change in the Netherlands, as winters are anticipated to become milder. A study by Grjibovski *et al.* [65] found no significant association between temperature and cardiovascular mortality in Astana, the second coldest capital in the world; they concluded that people living in such cold climates were just better adapted to cold (e.g., culture of wearing large volumes of winter clothes outdoors) than populations living in other regions. McMichael *et al.* [20] found that sensitivity to cold tended to be higher in warmer cities such a Bangkok compared to colder cities like Bucharest, although the factors contributing to this difference could not be explained. A European study by Healy *et al.* [49] found that in mid-latitude populations the steepness of the relationship between cold temperatures and mortality was inversely related to the average winter temperature. Likewise, Analitis [50] concluded that within Europe the cold effect was found to be greater in warmer (southern) cities. Hence, a scenario anticipating that the slope of the ERF for cold exposure becomes steeper (compared to the baseline) could perhaps be an appropriate adaptation assumption to consider for the Netherlands. Of course, many developments that have lowered our vulnerability to cold, such as improved housing, increasing overall living standards and improvements in health care, are not likely to reverse. However, physiological and behavioural adaption (e.g., wearing less protective clothing in winter [48]) might occur as a result of milder winters. We, therefore, included the assumption that vulnerability to cold will increase with a hypothetical 10% in our adaptation scenarios.

Some limitations must be acknowledged. Observed temperature–mortality relationships depend to some degree on the statistical methods used to derive them, such as the lag structures used and seasonal controls [20,66]. We need to recognize the current discussion about the causal mechanisms behind the widely observed association between cold and mortality; the importance of the influence of seasonal factors other than temperature on winter mortality have been stressed and the potential for decreasing cold-related deaths due to warmer winters can be overestimated unless the effects of influenza and season are taken into account [6,67,68]. Recent studies [18,21,50] reporting associations between cold temperatures and mortality often assume longer time lags (e.g., up to 3–4 weeks) and control for season. The study by Hajat *et al.* [21], for example, modelled cold impacts using temperatures lagged by up to 28 days, while controlling for other seasonal patterns and influenza. However, Kinney *et al.* [68] emphasize the difficulty of using long time lags, as long moving averages of temperature are highly correlated with seasonal mortality patterns. In an effort to control for season, they analyzed winter mortality within monthly strata across multiple cities. According to their results, daily mortality was not strongly influenced by same day temperature. Using the average temperature over the five preceding days, the cold effect was somewhat larger. However, using longer time lags is not possible using monthly strata, demonstrating the difficulty of resolving longer lagged temperature effects from season [18]. Hence, the model used to estimate the baseline Dutch exposure–response function [37] may have overestimated the cold effect, which is sensitive to methods of adjustment for unmeasured seasonal influences and cumulative lag assumptions. Moreover, if the widely observed association between mortality and cold would indeed be largely complicated by other factors, the assumption that the sensitivity to cold could increase as winters become milder might perhaps be less plausible.

We also need to recognize that, although the study by Huynen *et al.* [37] estimating the Dutch ERF between mortality and temperature was in many respects advanced for its time (e.g., the implicit non-linear distributed lag model), more advanced approaches are currently used to model multi-lag nonlinear temperature mortality relationships [66,69]. Another methodological consideration is the inclusion of demographic change. The current study accounts for the anticipated increase in overall mortality by 2050. However, the analyses was not performed for different age groups, while the proportion of the elderly—found to be among the most vulnerable to temperature-related mortality effects [6]—is expected to increase. Thus, it can be argued that our results provide a conservative estimate of possible future changes in temperature-related mortality.

## 5. Conclusions

Given the uncertainties involved in estimating future heat- and cold-related mortality in view of a changing climate and subsequent adaptation, our analysis does not pretend to yield precise results or definitive conclusions about the temperature-related mortality effects of climate change in the Netherlands. Its main objective is to provide an order-of-magnitude estimate of the effects, acknowledging the uncertainties involved and communicating the assumptions made in an explicit way. In doing so, we account for the interplay between global warming, changing air circulation patterns in Western Europe, possible adaptation pathways and increasing overall mortality. Our results show that the model outcomes are not only highly dependent on climate scenarios, but also on assumptions about future adaptation.

Due to the long-term nature of many global environmental health challenges, integrated environmental health impact assessment requires foresight methodologies. The adaptation scenarios included in our assessment present a sample from a wide variety of possibilities in order to start thinking about the impact of future adaptation. Currently, that are no guidelines for scenario-based future research on temperature-related mortality and the methods used for projections are still in their early stages and have limitations [19]. It is uncertain how much adaptation may mitigate the effects on human health [6], and there is currently no consensus on how to account for adaptation in integrated health impact assessment models. However, climate change and adaptation to climate change are interlinked processes, which should simultaneously be taken into account in an integrated health impact assessment. Assuming that the temperature–mortality relationship will remain the same might be equally plausible as any other adaptation scenario. Huang *et al.* [19] conducted a review of studies regarding future heat-related mortality; about seven of the 14 selected studies included some form of adaptation in their projections, but only one [47] was explicitly based on more than one adaption scenario.

Hence, we stress that a better understanding of the impact of various plausible adaptation assumptions is required to advance future research. Future assessments of heat- and cold-related mortality should continue to explore the impacts of different adaptation options, affecting both the optimum temperature level as well as plausible developments in the sensitivity to heat and cold. More extended research is required to improve our understanding of the modulating roles of such factors as housing quality, technology, local topography, urban design and behavioral factors, and to improve assessment of adaptive capacity to current and future climates [20]. For example, regional differences in vulnerability to heat and cold could be further examined in order to identify possible mortality effects of different adaptation scenarios. In doing so, the need to identify and track (future) uncertainties in the assessment process, and to report them in a meaningful way as part of the assessment results, is evident from the outset [23]. This challenges health scientists and epidemiologists to extend their conventional methodological boundaries. Similar to the climate change community, they could embrace the use of scenarios as a tool to enhance our understanding of the implications of possible future developments. Thinking about what might happen under different future adaptation pathways can potentially play an important role in impact assessment studies in order to capture a plausible range of climate change impacts, which would usually be beyond the scope of more traditional forms of health impact assessment.

## Appendix

**Table A1:** Population attributable fractions of total mortality due to exposure to heat and cold, at baseline (1981–2010) and around 2050 (KNMI’14 climate scenarios), the Netherlands: model runs with and without adaptation scenarios.

Total Mortality				
Climate Scenario	Adaptation Scenario *	PAF_heat_	PAF_cold_	PAF_temperature_
Baseline (1981–2010)	n.a.	1.15%	8.90%	10.05%
KNMI’14 G_L_	no adaptation	1.66%	7.85%	9.51%
	I	1.11%	8.96%	10.07%
	II	1.50%	8.59%	10.09%
	III	1.00%	9.80%	10.81%
KNMI’14 G_H_	no adaptation	1.87%	7.49%	9.36%
	I	1.11%	9.00%	10.11%
	II	1.69%	8.20%	9.89%
	III	1.00%	9.85%	10.85%
KNMI’14 W_L_	no adaptation	2.18%	6.83%	9.02%
	I	1.04%	8.92%	9.96%
	II	1.97%	7.48%	9.46%
	III	0.94%	9.76%	10.70%
KNMI’14 W_H_	no adaptation	2.52%	6.56%	9.08%
	I	1.14%	8.91%	10.06%
	II	2.28%	7.19%	9.46%
	III	1.03%	9.75%	10.79%

***** Adaptation scenario I = shift in optimum temperature; Adaptation scenario II = changing sensitivity to heat and cold; Adaptation scenario III = shift in optimum temperature as well as changing sensitivity to heat and cold.

**Table A2:** Population attributable fractions of cardiovascular mortality due to exposure to heat and cold, at baseline (1981–2010) and around 2050 (KNMI’14 climate scenarios), the Netherlands: model runs with and without adaptation scenarios.

Cardiovascular Mortality				
Climate Scenario	Adaptation Scenario *	PAF_heat_	PAF_cold_	PAF_temperature_
Baseline (1981–2010)	n.a.	0.79%	10.85%	11.64%
KNMI’14 G_L_	no adaptation	1.14%	9.58%	10.72%
	I	0.77%	10.92%	11.68%
	II	1.03%	10.47%	11.50%
	III	0.69%	11.93%	12.62%
KNMI’14 G_H_	no adaptation	1.29%	9.14%	10.43%
	I	0.76%	10.97%	11.73%
	II	1.16%	10.00%	11.17%
	III	0.69%	11.99%	12.68%
KNMI’14 W_L_	no adaptation	1.51%	8.35%	9.85%
	I	0.71%	10.87%	11.59%
	II	1.36%	9.14%	10.49%
	III	0.64%	11.88%	12.53%
KNMI’14 W_H_	no adaptation	1.74%	8.01%	9.76%
	I	0.79%	10.86%	11.65%
	II	1.57%	8.77%	10.34%
	III	0.71%	11.87%	12.58%

***** Adaptation scenario I = shift in optimum temperature; Adaptation scenario II = changing sensitivity to heat and cold; Adaptation scenario III = shift in optimum temperature as well as changing sensitivity to heat and cold.

**Table A3:** Population attributable fractions of respiratory mortality due to exposure to heat and cold, at baseline (1981–2010) and around 2050 (KNMI’14 climate scenarios), the Netherlands: model runs with and without adaptation scenarios.

Respiratory Mortality				
Climate Scenario	Adaptation Scenario *	PAF_heat_	PAF_cold_	PAF_temperature_
Baseline (1981–2010)	n.a.	5.05%	29.11%	34.16%
KNMI’14 G_L_	no adaptation	7.25%	26.04%	33.29%
	I	4.90%	29.28%	34.18%
	II	6.58%	27.05%	34.44%
	III	4.45%	31.62%	36.07%
KNMI’14 G_H_	no adaptation	8.14%	24.98%	33.12%
	I	4.89%	29.40%	34.29%
	II	7.40%	27.05%	34.44%
	III	4.43%	31.76%	36.19%
KNMI’14 W_L_	no adaptation	9.45%	22.99%	32.44%
	I	4.59%	29.17%	33.75%
	II	8.60%	24.92%	33.52%
	III	4.16%	31.51%	35.67%
KNMI’14 W_H_	no adaptation	10.86%	22.15%	33.00%
	I	5.04%	29.15%	34.19%
	II	9.88%	24.02%	33.90%
	III	4.58%	31.48%	36.06%

***** Adaptation scenario I = shift in optimum temperature; Adaptation scenario II = changing sensitivity to heat and cold; Adaptation scenario III = shift in optimum temperature as well as changing sensitivity to heat and cold.

**Table A4:** Baseline and future number of deaths attributable to heat (M_heat_) and cold (M_cold_), at baseline (1981–2000) and in 2050 (KNMI’14 climate scenarios), the Netherlands: model runs with and without adaptation scenarios.

Climate Scenario	Adaptation Scenario **	Number of Deaths Attributable to Heat (% of Baseline)	Number of Deaths Attributable to Cold (% of Baseline)
baseline		1511 (100%)	11,727 (100%)
2050 no climate change *****	n.a.	2299 (152%)	17,846 (152%)
KNMI’14 G_L_ *****	no adaptation	3329 (220%)	15,733 (134%)
	I	2230 (148%)	17,965 (153%
	II	3002 (199%)	17,226 (147%)
	III	2011 (133%)	19,657 (168%)
KNMI’14 G_H_ *****	no adaptation	3752 (248%)	15,020 (128%)
	I	2222 (147%	18,052 (154%)
	II	3384 (224%)	16,447 (140%)
	III	2004 (133%)	19,753 (168%)
KNMI’14 W_L_ *****	no adaptation	4380 (290%)	13,699 (117%)
	I	2083 (138%)	17,886 (153%)
	II	3952 (262%)	15,007 (128%)
	III	1879 (124%)	19,572 (167%)
KNMI’14 W_H_ *****	no adaptation	5061 (335%)	13,149 (112%)
	I	2295 (152%)	17,872 (152%)
	II	4567 (302%)	14,406 (123%)
	III	2070 (137%)	19,556 (167%)

***** Only accounting for the anticipated increasing trend in annual mortality up to 2050 (2050 model runs applying baseline attributable fractions to future number of annual deaths); ****** Adaptation scenario I = shift in optimum temperature; Adaptation scenario II = changing sensitivity to heat and cold; Adaptation scenario III = shift in optimum temperature as well as changing sensitivity to heat and cold.
